# Treating sex and gender differences as a continuous variable can improve precision cancer treatments

**DOI:** 10.1186/s13293-024-00607-1

**Published:** 2024-04-15

**Authors:** Wei Yang, Joshua B. Rubin

**Affiliations:** 1 Department of Genetics, Washington University School of Medicine, St. Louis, MO 63110 USA; 2 Department of Pediatrics, Washington University School of Medicine, St. Louis, MO 63110 USA; 3 Department of Neuroscience, Washington University School of Medicine, St. Louis, MO 63110 USA

**Keywords:** Sex and gender differences, Cancer, Hallmark pathways, Cell cycle regulation, Inflammation/immunity, Bayesian analyses, Personalized medicine

## Abstract

**Background:**

The significant sex and gender differences that exist in cancer mechanisms, incidence, and survival, have yet to impact clinical practice. One barrier to translation is that cancer phenotypes cannot be segregated into distinct male versus female categories. Instead, within this convenient but contrived dichotomy, male and female cancer phenotypes are highly overlapping and vary between female- and male- skewed extremes. Thus, sex and gender-specific treatments are unrealistic, and our translational goal should be adaptation of treatment to the variable effects of sex and gender on targetable pathways.

**Methods:**

To overcome this obstacle, we profiled the similarities in 8370 transcriptomes of 26 different adult and 4 different pediatric cancer types. We calculated the posterior probabilities of predicting patient sex and gender based on the observed sexes of similar samples in this map of transcriptome similarity.

**Results:**

Transcriptomic index (TI) values were derived from posterior probabilities and allowed us to identify poles with local enrichments for male or female transcriptomes. TI supported deconvolution of transcriptomes into measures of patient-specific activity in sex and gender-biased, targetable pathways. It identified sex and gender-skewed extremes in mechanistic phenotypes like cell cycle signaling and immunity, and precisely positioned each patient’s whole transcriptome on an axis of continuously varying sex and gender phenotypes.

**Conclusions:**

Cancer type, patient sex and gender, and TI value provides a novel and patient- specific mechanistic identifier that can be used for realistic sex and gender-adaptations of precision cancer treatment planning.

**Supplementary Information:**

The online version contains supplementary material available at 10.1186/s13293-024-00607-1.

## Abstract (plain language summary)

Some efforts to improve cancer therapy involve the idea of personalizing treatments to who a patient is and how their cancer operates. Personalizing treatment can involve straighforward features like a patient’s age, family cancer history, personal disease and surgical histories, as well as more complex features like analysis of their specific cancer’s mechanisms of growth and spread throughout the body. One glaring omission in common personalization schemes is the sex and gender of the patient. While patient sex and gender is known to substantially affect cancer rates and response to treatment, we do not yet use this information in treatment planning. There are multiple reasons for this but among them is that we tend to think about sex and gender as an either/or categorization. You are either a male/man or a female/woman. This is not accurate as there are many variables that contribute to who an individual is as a male/man or female/woman. This variability is a challenge to incorporating these features into personalized treatment planning. Here, we have developed a method to address this challenge. It is our great hope that this will enable the use of this critically important element of personalization in cancer treatment planning and improve survival rates for all patients.

**Supplementary Information:**

The online version contains supplementary material available at 10.1186/s13293-024-00607-1.

## Highlights

**•** Sex and gender effects on cancer incidence and survival can be more realistically addressed by treating sex and gender as continuous, rather than categorical, variables.
**•** Bayesian analysis of whole cancer transcriptomes can uniquely place individual patients along a continuum of sex and gender effects.
**•** Locations along this continuum are associated with specific cancer hallmark pathway activation profiles.
**•** These patient-specific pathway profiles may be used for treatment stratification.
**•** This approach can enhance the personalization of cancer treatments.

**Supplementary Information:**

The online version contains supplementary material available at 10.1186/s13293-024-00607-1.

## Background

Significant sex and gender differences in cancer incidence and mortality are recognized to be the norm with most shared cancers exhibiting male to female incidence ratios ranging from 1.26:1 to 4.86:1 [1]. Recent analysis of over 14 million cases from the Cancer Registry, representing 99.9% of the cancer population of the United States confirmed an overall predominance of male cancer cases [2]. An accompanying analysis of survival data from 3.7 million cases in the Surveillance, Epidemiology, and End Results (SEER) database, representing approximately 28% of the cancer population, confirmed that mortality rates are higher for males compared to females [2]. These clinically important sex and gender differences are concordant with described sex differences in cell biology including response to genotoxic stress [3, 4], DNA repair [5, 6], mutational burden and oncogenic mechanisms [7, 8], metabolism [9, 10], and cell cycle regulation [11–13], as well as in systems biology including: immunity [14], metabolism [15, 16], tissue repair [17, 18], and longevity [19, 20]. This suggests that therapies for all cancer patients may be advanced by a realistic translation of sex and gender differences into clinical practice.

While our awareness of sex differences in cellular and systems-wide biology continually advances, an obstacle to successful translation of the work to date, is our incomplete understanding of how the genetic, epigenetic, and hormonal foundations of sexual differentiation mechanistically interact with cancer hallmark pathways during tumorigenesis, progression, and response to treatment. A second obstacle is the nature of sex and gender differences. Most sex and gender differences are not dichotomous or dimorphic. Instead, most sex and gender differences are more akin to height, a complex trait that varies continuously between the shortest females and the tallest males, and is intermediate for most people [21]. Thus, investigating sex and gender effects in cancer is complicated by the varying and age-dependent interactions between chromosomal (XX vs. XY) and gonadal sex (ovaries vs. testes), imprinting and other epigenetic effects during sexual differentiation [22], the epigenetics of life-histories [23] and the varying cellular, tissue, and systemic mechanisms underlying individual cancer phenotypes [1]. A third obstacle are the recognized ambiguities and inequities in current usage of terms like male, female, men, and women. In this study, we use female and male as aggregate terms to represent the entangled nature of sex and gender [24]. We recognize that this may perpetuate a false dichotomy, but as our goal is to change clinical practice now, we are constrained by the current classifications of sex and gender in clinical data. Furthermore, It is important to stress that many cancer-relevant traits such as, growth regulation, metabolism, and immunity, aggregate around male and female poles. Thus, while not complete or wholly accurate, categorical sex contains a lot of information about sex effects on the range of human phenotypes in health and disease. They provide useful points of reference.

We sought a method by which to identify mechanisms underlying cancer phenotypes that varied as a function of sex and gender. We expected this would augment the traditional binary classifications by integrating them with individual patient-based transcriptome data. To do this, we calculated a patient-specific Transcriptomic Index (TI) value based on a Bayesian Nearest Neighbor (BNN) analysis that quantifies local differences within the transcriptome-similarity space, and precisely located individuals relative to the female (smallest TI values) and male (highest TI values) poles. We determined that most cancer diagnostic groups exhibit transcriptomic variance that correlates to sex and gender and that cell cycle regulation and immunity/inflammation are the pathways most frequently associated with the male and female poles, respectively. Further, we identified differing mechanisms associated with midrange TI values in female and male patients. We conclude that even when males and females exhibit overlapping phenotypes, the mechanisms underlying that phenotype can differ. This is consistent with published analyses demonstrating that even when genes are equally expressed in males and females, they can exhibit different correlations to cancer mechanisms, treatment responses, and survival [25, 26]. We expect that the TI approach will advance laboratory and clinical research into sex and gender effects and provide a paradigm for using an individual’s entire transcriptome for planning their individualized cancer treatment.

## Materials and methods

### Inferring Transcriptomic Index (TI) using bayesian nearest neighbors

We downloaded the TCGA pan-cancer transcriptome data (gene expression RNAseq - Batch effects normalized mRNA data) from https://pancanatlas.xenahubs.net, the Kids First neuroblastoma data from dbGaP (https://www.ncbi.nlm.nih.gov/projects/gap/cgi-bin/study.cgi?study_id=phs001436.v1.p1) and the Children’s Brain Tumor Network brain tumor data from https://cbtn.org. Sample sizes and number of genes are listed in Table 1. We excluded non-malignancies, cancer types with highly skewed numbers of male or female cases, and those cancers with < 45 cases in the datasets. The remaining 26 adult and 4 pediatric cancers have sample sizes ranging from 45 to 572, with male samples comprising 27.2–84.2% of each cancer type. The total cases examined were 8370 (4927 Males (58.9%), 3443 Females (41.1%)).

Next, we sought to define the Transcriptomic Index (TI) as the Bayesian posterior probability of predicting a patient’s sex from the nearest neighbors based on transcriptomic Euclidean distances. An advantage of the Bayesian Nearest Neighbor (BNN) algorithm is that it can infer “breakpoints” between local groupings of nearest neighbors and estimate individual TI values for any transcriptome along a continuous spectrum of values as a Bayesian posterior probability using that transcriptomes’ local neighbors [27].

Following the notations from [27] we denote the target point for predicting patient sex as ${x}_{\tau }$, and the available training data as ${x}_{0},{x}_{1},\cdots ,{x}_{\tau -1}$, ordered by distance to ${x}_{\tau }$, with ${x}_{0}$ being the most distant point from the target. Over a partition ρ of the ordered data points, assume the data is independent and identically from a Bernoulli distribution $P\left(x|\rho \right)\sim Ber\left(\theta \right)$, i.e., $P\left({x}_{i}=1|i\in \rho \right)=\theta$ and $P\left({x}_{i}=0|i\in \rho \right)=1-\theta$, with $x=1$ indicating the sample being male and $x=0$ indicating female. The conjugate prior of the Bernoulli distribution is a Beta distribution, $\theta \sim Beta\left(\alpha ,\beta \right)$. Moreover, to model the partition $\rho$, we use ${k}_{i}$ to denote the number of neighbors in the same partition before sample $i$ when moving from ${x}_{0}$ towards ${x}_{\tau }$. Starting from the farthest point, we have

$$p\left({k}_{0}=0\right)=1$$

When observing a new datum and moving closer towards the target, we either have a breakpoint and start with a new partition with a certain probability ${p}_{\gamma }$, or extend the previous partition by 1 at a probability of $1-{p}_{\gamma }$, i.e.,

$$p\left({k}_{i}=0|{k}_{i-1}\right)={p}_{\gamma }$$

$$p\left({k}_{i}={k}_{i-1}+1|{k}_{i-1}\right)={1-p}_{\gamma }$$

With the above assumptions, we can recursively calculate the joint probability $p\left({k}_{i}, {x}_{0},\cdots ,{x}_{i}\right)$ starting from ${x}_{0}$ as,

$$\begin{aligned}& p\left({k}_{i}={k}_{i-1}+1, {x}_{0},\cdots ,{x}_{i}\right)=\\&\quad{p}\left({k}_{i-1}, {x}_{0},\cdots ,{x}_{i-1}\right)p\left({x}_{i}|{k}_{i},{x}_{0},\cdots ,{x}_{i-1}\right){p}_{\gamma}\end{aligned}$$

$$\begin{aligned}& p\left({k}_{i}=0, {x}_{0},\cdots ,{x}_{i}\right)\\&=p\left({x}_{i}|{k}_{i}=0\right)\sum _{{k}_{i-1}}p\left({k}_{i-1}, {x}_{0},\cdots ,{x}_{i-1}\right)\left(1-{p}_{\gamma }\right)\end{aligned}$$

In these equations, we can have $p\left({x}_{i}|{k}_{i},{x}_{0},\dots , {x}_{i-1}\right)$ and $p\left({x}_{i}|{k}_{i}=0\right)$ directly calculated from the Bernoulli distribution. After estimating the joint probabilities, we can easily calculate $p\left({k}_{i}| {x}_{0},\cdots ,{x}_{i}\right)$ and therefore the final Bayesian posterior probability $p\left({x}_{\tau }| {x}_{0},\cdots ,{x}_{\tau -1}\right)$by integrating over the distribution of the number of neighbors ${k}_{\tau }$. More details of the algorithm for estimating the model could be found from [27]. When applying the above Bayesian model, we used priors $\alpha =10$ and $\beta =10$ for the Beta priors, and ${p}_{\gamma }=0.05$ for the breakpoint probability.

The TI value, calculated using BNN posterior probability, directly measures local sex differences within the transcriptome-similarity space. A value close to 1 indicates enrichment with male samples, while a value close to 0 indicates enrichment with female samples. In our analysis, TI values were calculated separately for each cancer type.

### Downstream analysis

After estimating TI for each patient, the association between TI and gene expression was assessed. Genes with expression positively associated with TI values were identified as male - skewed genes, while genes with expression negatively associated with male TI values were identified as female - skewed genes. Linear regression was used for testing gene associations and variable correlations. Enrichment of male and female skewed genes were tested in MSigDB hallmark gene sets [28] using one-sided Fisher’s exact test. Multiple test corrections were performed using the Benjamini-Hochberg FDR Procedure for gene association test and pathway analysis. All measures in the analysis were taken from distinct samples of the involved subjects.

### Variability in male-to-female ratios across cancer types

To account for the variability in male-to-female ratios across different cancer types, we initially implemented a weighted version of the BNN algorithm for estimating TI indexes. We assigned weights inversely proportional to the male-to-female ratios for male and female samples within each cancer type. This ensured that the Bayesian algorithm was provided with balanced overall weights for both male and female samples. Furthermore, in the comparative analysis across cancer types, instead of directly comparing inferred TI values, we aggregated the results and conducted comparisons at the level of significant genes and pathways associated with alterations in TI indexes.

## Results

We previously applied the Joint and Individual Variance Explained (JIVE) algorithm to decompose male and female glioblastoma transcriptome data into components shared among males and females, and those unique to each sex [26]. This approach identified “sex-specific” gene expression patterns and sex-based molecular subtypes of GBM. While informative, the JIVE approach has limited clinical utility because it requires categorical assignment of gene expression to “male-specific”, “female-specific”, and shared components. “Sex” as a categorical variable has important but limited value when investigating the spectrum of sex and gender differences or attempting to stratify individual patients for sex and gender-informed treatments. Thus, we sought a method for generating individual patient-specific values along an axis that traversed between female and male cancer transcriptional sex and gender “poles”.

**Fig. 1:**
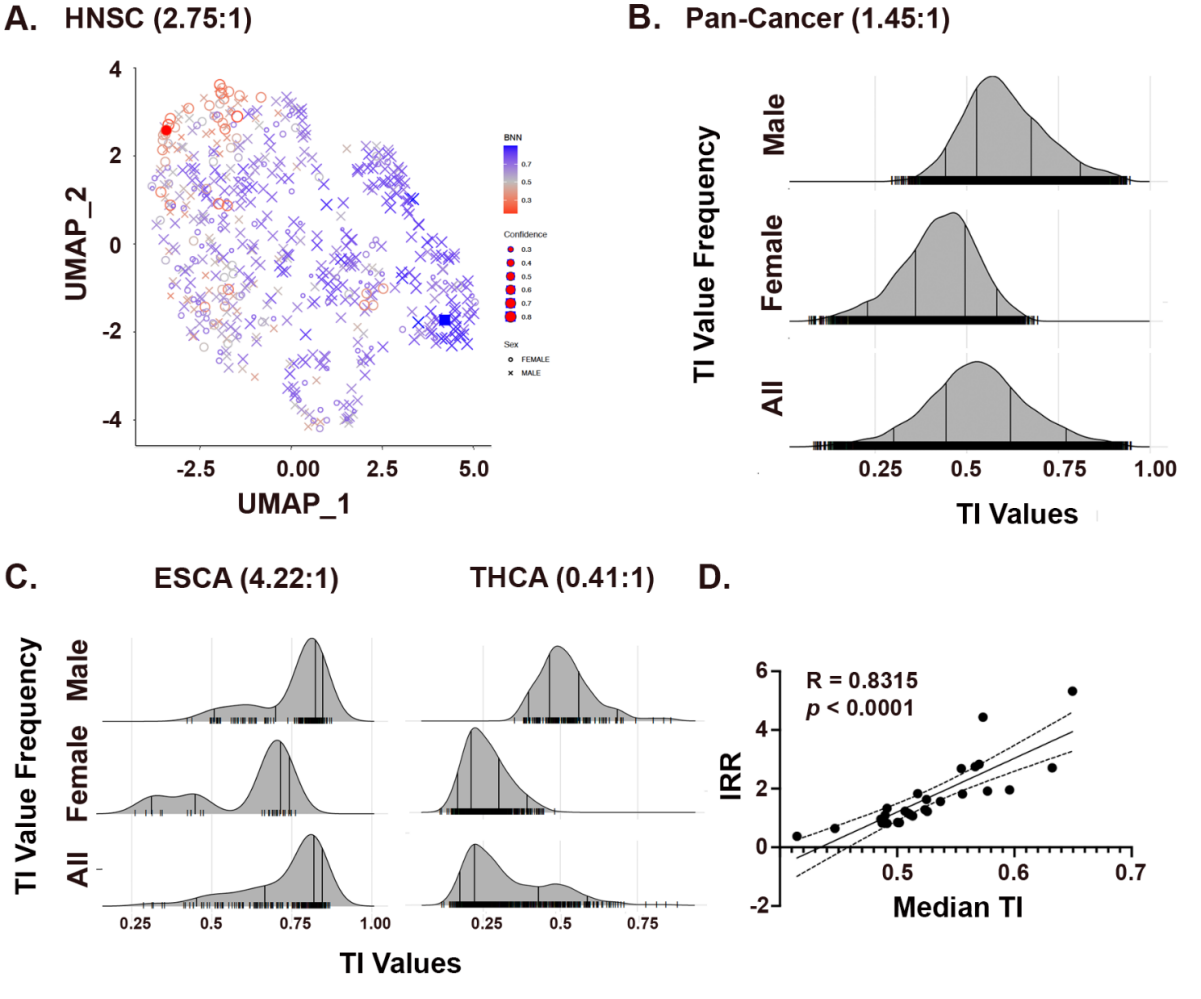
Cancer transcriptomes exhibit skewing by sex and gender. (**A**) UMAP of 566 HNSC transcriptomes clustered by similarity. Male: Female Incidence rate ratio is shown. Male (blue X’s) and female (Red circles) distribute throughout the transcriptional space. Local enrichments for male and female transcriptomes were recognized and quantified to define female (filled red circle) and male (filled blue square) poles of gene expression. TI value is color-coded and confidence in the posterior probability is indicated by symbol size as indicated. (**B**) Ridge plots of the TI value distributions for Male, Female, and All patients from the PANCAN data (7881 total, 4668 M, 3213 F., M: F IRR = 1.45:1), with vertical lines indicating the 5%, 25%, 75% and 95% quantiles, respectively. (**C**) Ridge plots for TI population distributions for Esophageal Carcinoma (ESCA, M/F IRR = 4.22) and Thyroid Carcinoma (THCA, M/F IRR = 0.41) illustrate the correlation between IRRs and median TI values for the 26 adult cancers. (**D**) Regression analysis of IRR vs. Median TI values. Shown is the best fit and 95% confidence intervals. R and *p* values are shown

We created UMAPs from the transcriptomes of each cancer based on similarities in gene expression. The head and neck squamous carcinoma (HNSC) UMAP, illustrates the process of identifying poles in the data (Fig. 1A). We discovered local areas in the UMAP where samples were primarily male (blue exes) or female (red circles). We quantified the local sex and gender enrichment in the transcriptome-similarity space using the TI values as described in the methods section, and identified the most skewed regions as poles (filled red circle and filled blue square) to detect skewing in cancer transcriptomes.

We derived TI values for all individual cases and median TI values in every cancer population separately (Supplemental Fig. 1). Then, we combined all 7881 adult TI values to create a pan-cancer TI population distribution (Fig. 1B). As can be seen, the female and male values are skewed. Further, cases with TI values below 0.25 are exclusively female while only males have values above 0.75. These cases represent the female and male population poles, respectively. It is also clear from the data that a large fraction of the cancer population possesses TI values between 0.25 and 0.75. We can expect that as female cases approach TI values of 0.5, they represent a changing balance between pole effects that will mechanistically differ from those in male cases approaching the same TI value.

As expected, median TI values for each cancer type positively correlated with their incidence rate ratios (IRR, M:F) as calculated from these datasets. As illustrated in Fig. 1C, esophageal carcinoma (IRR = 4.22) and thyroid carcinoma (IRR = 0.41) exhibit median TI values of (0.65) and (0.41), respectively. Similarly, the other cancers with IRRs of less than 1 (sarcoma, adrenocortical carcinoma, diffuse large B cell lymphoma, thyroid carcinoma) exhibit median TI values of less than 0.50 (Supplemental Table 1). Regression analysis of IRR versus median TI identified a significant correlation between the two (Fig. 1D). Thus, TI value distributions are concordant with sex and gender differences in individual cancer IRRs. Importantly, TI value indicates that many male individuals with IRR < 0.5 cancers exhibit individual TI values that are shifted towards the female pole and that many female individuals with IRR > 0.5 cancers exhibit individual TI values that are shifted towards the male pole. This does not mean that some female cancers are “male-like”, and some male cancers are “female-like.” Instead, it highlights the shortcomings of these classifications and ambiguities that can arise with their use. The TI value simply describes a phenotype like a taller than average female or a shorter than average male.

These data indicate that individual transcriptomic variation across cancer types retains signatures of sex and gender, and suggest that sexual differentiation may have foundational effects on cancer phenotypes. Thus, we next sought to identify the genes and pathways that define the high and low TI poles. We did so by looking for consistency in sex and gender-skewed mechanisms across cancer types using the 7881 adult and in parallel, the 1069 pediatric cases. Those genes with the greatest effect on low and high TI values were identified by performing association analysis between TI and gene expression. Genes that were significantly (FDR < 0.05) associated with high TI were identified as “male-skewed genes”, while those negatively associated with high TI were identified as “female - skewed” (Supplemental Table 2).

**Fig. 2:**
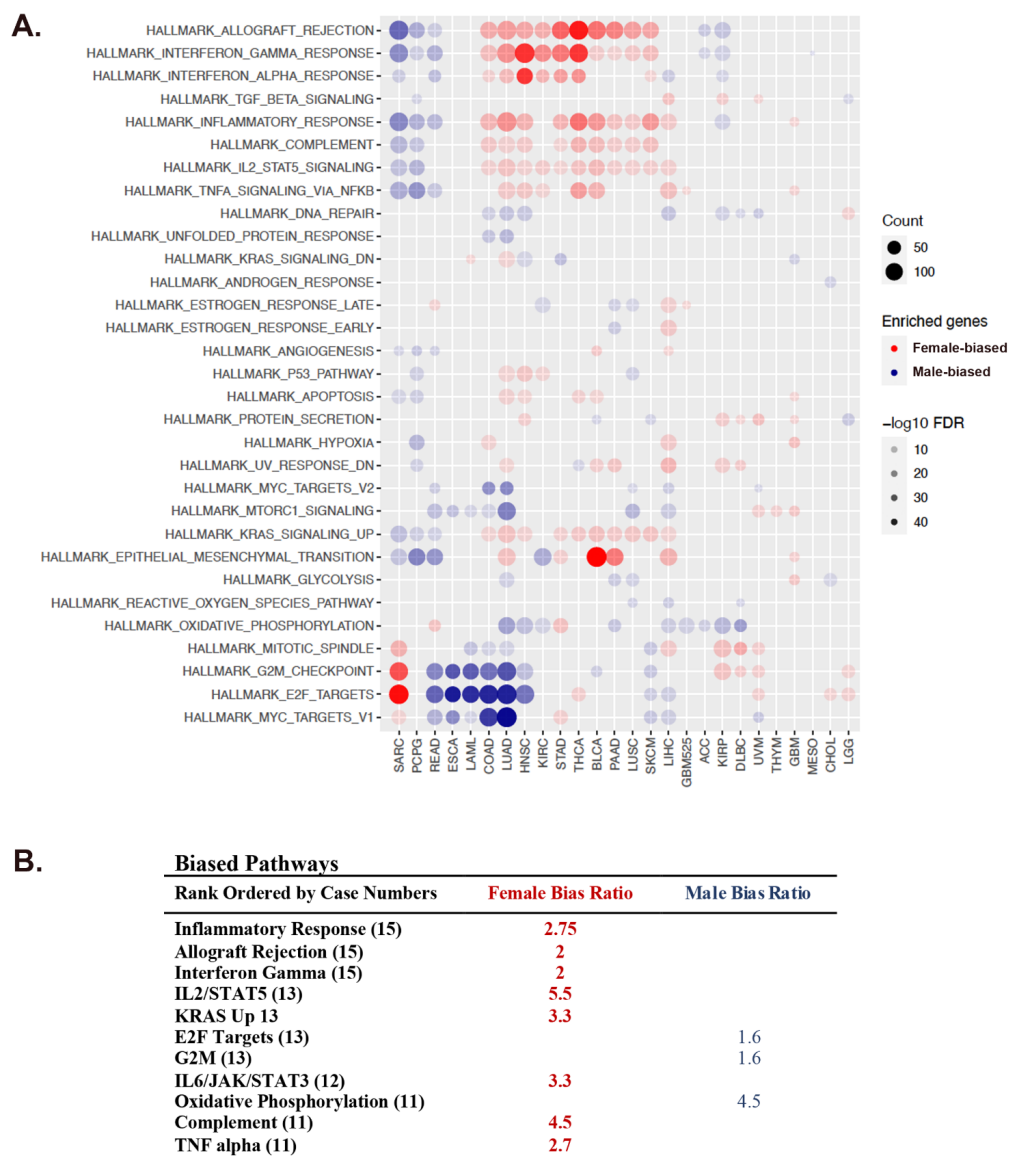
Most Cancers exhibit sex and gender - skewed hallmark pathway activation. (**A**) Genes with the greatest effect on low (female pole) and high (male pole) TI values were identified. Cancer Hallmark Pathway analysis of pole-associated genes revealed a predominant polarization pattern involving cell cycle regulation and oxidative phosphorylation at the male pole (blue circles) and multiple inflammatory/immunity pathways at the female pole (red circles). Gene counts (count) are symbolized by the size of the circles and False Discovery Rates (FDR) by the saturation of the fill as indicated in the legends. (**B**) Frequency of pathway skewing is listed in rank order of cases involved (in parentheses) and the ratio of involved female (red text) to male cases (blue text)

Cancer Hallmark Pathway analysis across cancer types indicated that most hallmark pathways [29] exhibit sex-skewing in gene expression and revealed several patterns of male versus female transcriptomic polarization (Fig. 2A and B). Seventeen of the 26 cancer types were enriched for genes involved in oxidative phosphorylation and/or cell cycle regulation at the male pole. Twelve of the 26 cancers were enriched for genes involved in inflammation and immunity at the female pole (Fig. 2A and B). Mesothelioma was the only cancer without evidence of transcriptomic polarization. Interestingly, sarcoma differed from the predominant polarization patterns such that male cases were enriched for inflammation/immunity signatures and female cases for cell cycle regulation. This emphasizes the need to interpret TI values within the context of cancer type and patient sex and gender.

These data indicate that varying degrees of sex and gender - correlated gene expression exist across cancer types and that a predominant shared pattern between multiple cancers involves skewed gene expression in cell cycle regulation versus inflammation/immunity pathways. This validates the TI approach as these pathways are known to be strongly sex and gender-biased in action [11–14]. The replication of these polarization patterns across cancer types provides a measure of cross-validation for the approach. Thus, we conclude that TI value can successfully localize individual cancer cases along axes that traverse between sex and gender poles in targetable mechanisms like cell cycle regulation and immunity/inflammation.

With some exceptions, *in utero* sexual differentiation results in outwardly recognizable male or female newborns, who differ in growth rates, immunity, metabolism, and disease risks, even prior to puberty and in the absence of circulating sex hormones. Thus, we hypothesized that if sexual differentiation patterns gene expression in cancer, we would also observe TI value sex-skewing in pediatric cancers. We analyzed two pediatric transcriptome datasets: the Gabriella Miller Kids First Pediatric Research Program (Kids First (KF)), which included 209 neuroblastoma patients and the Children’s Brain Tumor Network (CBTN), which included 865 patients comprised of 101 high grade glioma, 105 medulloblastoma, 79 ependymoma, and 214 low grade glioma cases (Table 1). From the KF data, 6330 male - skewed genes and 6089 female - skewed genes were identified (FDR < 0.05, Supplemental Table 3). From the CBTN data, 3063 male - skewed genes and 2062 female - skewed genes were identified (FDR < 0.05, Supplemental Table 4). There were 1126 shared male - skewed genes, and 742 shared female - skewed genes between the two datasets (both with *p* < 2.2e-16, Supplemental Table 5).

**Fig. 3:**
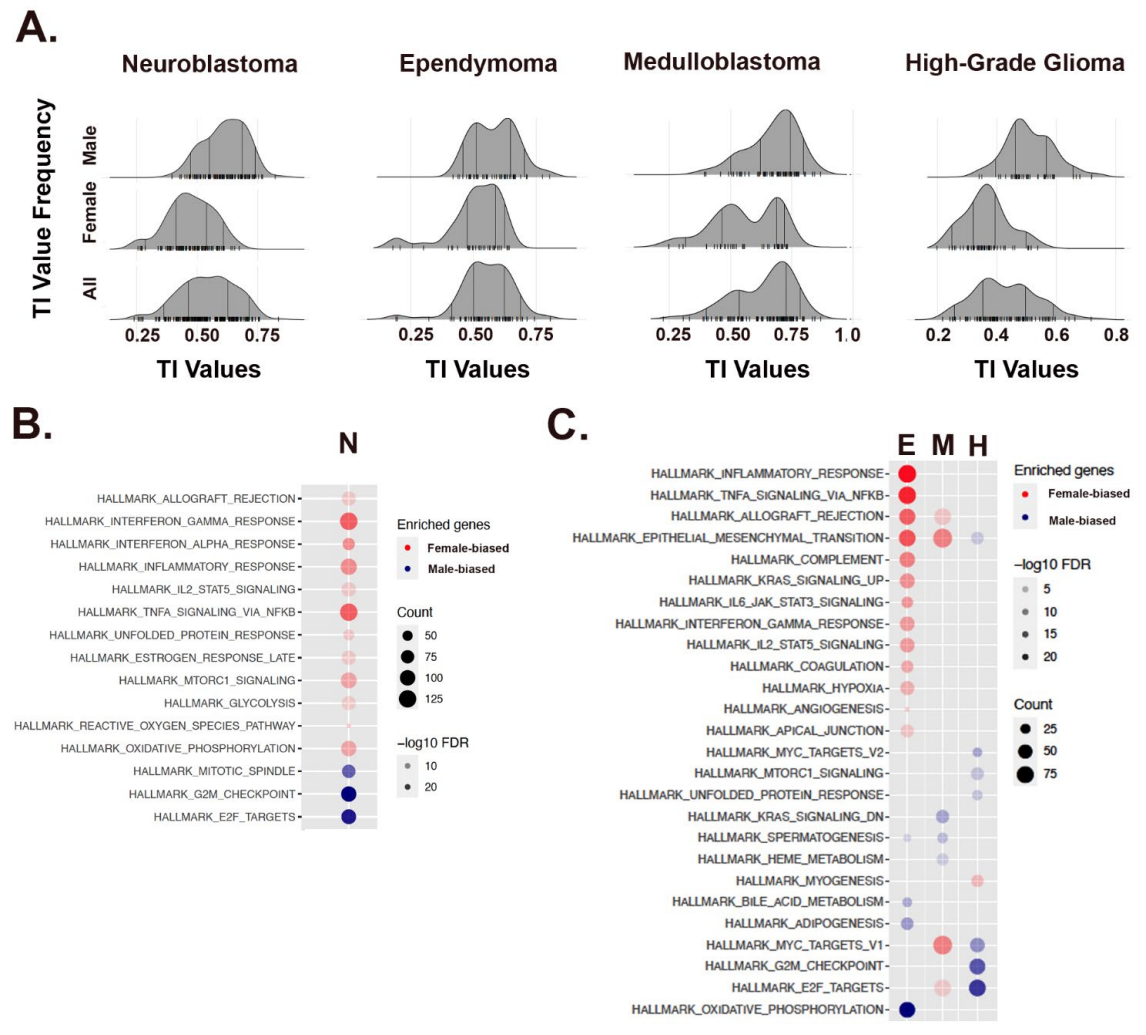
Pediatric neural tumors also exhibit sex and gender - skewed gene expression. (**A**) Ridge plots for neuroblastoma and the three most common malignant brain tumors of childhood (489 total, 259 M, 230 F., M: F IRR = 1.13:1) demonstrating sex -skewed TI population distributions, with vertical lines indicating the 5%, 25%, 75% and 95% quantiles, respectively. (**B)(C**) Cancer Hallmark Pathway analysis of those genes that exerted the greatest effects on the male and female poles for each cancer. A predominant polarization pattern is identified with inflammatory/immunity pathways associated with the female pole (red circles) and cell cycle regulatory pathways associated with the male pole (blue circles). Gene counts (count) are symbolized by the size of the circles and False Discovery Rates (FDR) by the saturation of the fill as indicated in the legends

Like the adult cancers, these pediatric cancers exhibited biased distributions of TI values (Fig. 3A, Supplemental Fig. 2). Neuroblastoma (IRR = 1.11) is strongly polarized and again, those cases with high TI values were enriched for cell cycle regulation while those associated with low TI values were enriched for inflammation and immunity (Fig. 3B). The CBTN brain tumor data includes the diverse tumor types common in pediatric neuro-oncology. We focused our analysis on the most common and malignant pediatric brain tumors. Ependymoma (IRR = 1.5) exhibited the strongest polarization. Again, low TI values were enriched for inflammation and immunity and oxidative phosphorylation was strongly correlated with high TI values (Fig. 3C). The most common malignant brain tumor of childhood is medulloblastoma (IRR: 1.8:1) [30]. The strongest association in medulloblastoma was between low TI value and cell cycle regulation, reminiscent of what was observed for adult sarcomas. In pediatric high-grade glioma (IRR ≈ 1), high TI values were strongly correlated with cell cycle regulation, while there were no distinct gene expression patterns associated with low TI value cases. Thus, like adult cancers, pediatric cancers exhibit sex and gender-skewed gene expression that varies in magnitude and involved pathways, with similarities between the pediatric and adult cancers in the associations between cell cyle regulation and oxidative phosphorylation with male cases, versus immunity and inflammation with female cases. Importantly, skewed gene expression and pathway activation are evident even when the incidence ratios of cancer types (e.g., pediatric high-grade glioma) near equivalence. Therefore, individuals with any cancer type can be more extensively phenotyped for personalized approaches to treatment using a TI analysis than without.

**Fig. 4:**
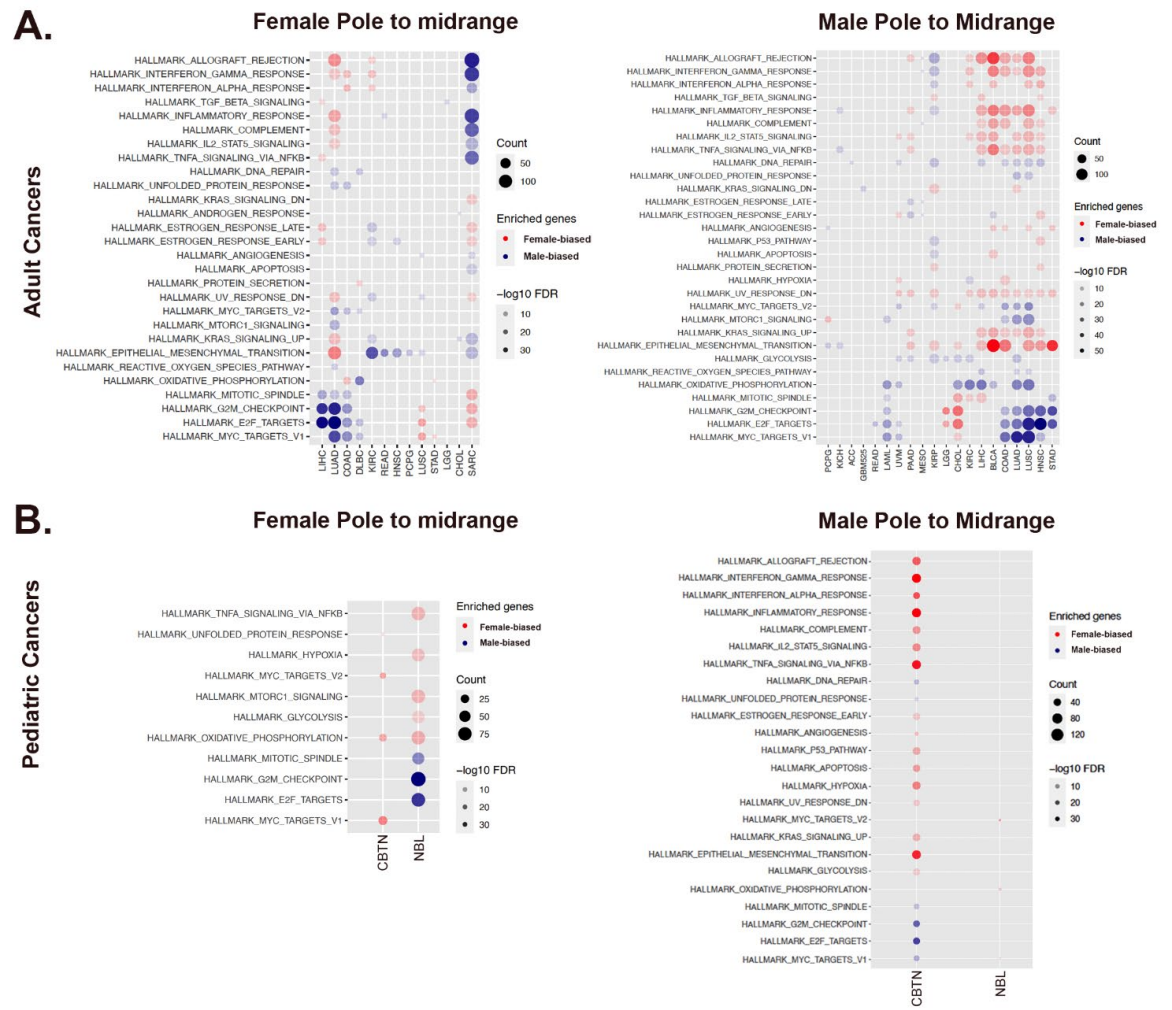
Mid-range TI values exhibit distinct pathway signatures relative to the sex and gender – defined poles. (**A**) (Left Panel) Heatmap of pathway activation signatures underlying changes in midrange TI values for female adult cancers relative to their pole. (Right Panel) Heatmap of pathway activation signatures underlying changes in midrange TI values for male adult cancers relative their pole. (**B**) (Left Panel) Heatmap of pathway activation signatures underlying changes in midrange TI values for pediatric female cancers relative to their pole. (Right Panel) Heatmap of pathway activation signatures underlying changes in midrange TI values for pediatric male cancers relative their pole. For all panels, changes in male (blue circles) and female (red circles) signatures are indicated. Gene counts (count) are symbolized by the size of the circles and False Discovery Rates (FDR) by the saturation of the fill as indicated in the legends. Only cancer types and pathways with significant change are shown

Cancer patients with extremes of high and low TI value might be approachable with something akin to sex and gender-specific treatments. However, TI values for most patients lie between the poles. Therefore, we expected that their transcriptomes would exhibit both female- and male- skewed components. We hypothesized that for female cases, translation along the TI axis from < 0.25 to midrange values would involve decreased female - skewed effects and/or increased male - skewed effects. We predicted that the opposite would be true for male cases with midrange TI value compared to those with TI > 0.75. If this proved to be the case, we expected TI could serve as a tool for stratification for sex and gender – informed treatments, even for those of differing sex and gender with identical TI value. To address this hypothesis, we compared the PANCAN transcriptomes of all cases with midrange TI value to those with transcriptomes closer to their respective poles. We then performed pathway analysis to determine which pathways were altered relative to the poles. Several clear patterns of change in different cancer types emerged. Across cancer types, most female cases, exhibited a loss of the inflammatory/immunity signatures (Fig. 4A). Female cases of four cancer types (LIHC, LUAD, COAD, DLBC) exhibited a gain in cell cycle regulatory signature. Male cases of seven cancer types (PRAAD, KIRC, LIHC, BLCA, COAD, LUAD, LUSC), exhibited a clear increase in the inflammation/immunity signature (Fig. 4A). There were mixed patterns of gains and losses of the other “pole-defining” pathways, such as cell cycle regulation and oxidative phosphorylation, across cancer types. In contrast, female sarcoma cases with midrange TI value cases exhibited gains in female - skewed cell cycle regulatory and male - skewed inflammation/immunity patterns of gene expression. Midrange male sarcoma cases exhibited no significant change in these pathways. Finally, several cancer type-specific changes in epithelial-to-mesenchymal transition (EMT) and key intracellular signaling pathways such as MYC, MTORC1, or KRAS, occurred in female and male cases with midrange TI values. Together, these data emphasize the potential of this approach for identifying sex and gender-skewed actions in targetable pathways, even for those with overlapping mid-range TI value.

We performed the same analysis in the pediatric datasets. Like the PANCAN analysis, translation away from respective poles to midrange TSI values occurred concomitantly with a shift in pole - defining pathway involvement (Fig. 4B). For increased power, we combined all malignant CBTN brain tumor cases for this analysis. We found that midrange TI value female cases exhibited decreased MYC targets (V1 and V2) and oxidative phosphorylation. Male CBTN cases with midrange TI value, exhibited significant changes in almost all hallmark pathways with gains in both the female - skewed inflammatory signature and loss of the male - skewed cell cycle and oxidative phosphorylation signatures.

In neuroblastoma, female midrange TI cases exhibited a strong acquisition of a male - skewed cell cycle regulatory signature as well as decreased female - skewed inflammation/immunity and metabolism signatures (Fig. 4B). No skewed pathway signature changes were detectable in the male midrange TI cases. Together, these results support the hypothesis that midrange TI value can be associated with different molecular pathway activation profiles in female and male individuals with different cancer diagnoses. Thus, even when similar in transcriptomic phenotype, particular subsets of female and male cancer patients may benefit from sex and gender-informed therapies.

## Discussion

Sex and gender effects in cancer incidence, treatment, and survival are commonplace [2]. Like any significant difference in cancer phenotypes, understanding the mechanistic basis for sex and gender effects holds promise for improving outcomes for all. Translating sex and gender-science to cancer treatment is complicated by the nature of sex and gender differences. Most sex and gender differences are not as dichotomous as a peacock’s tail. Instead, individuals are a unique blend of sex and gender actions, which change as a function of age. This suggests that the optimal translation of sex and gender differences in cancer biology will require recognizing that sex and gender are continuous and dynamic variables.

Here, we used a Transcriptomic Index to place an individual’s cancer transcriptome along sex and gender axes. This revealed a sex-informed view of their cancer biology and could be used to make predictions about pathways to target in their cancer. It is important to note that detecting the spectrum of sex-biased phenotypes required that we first impose the usual sex dichotomy. This was productive as many cancer-relevant pathways, such as growth regulation, metabolism, and immunity, aggregate around this dichotomy, and while not complete or wholly accurate, categorical sex contains a lot of information about sex effects on the range of human phenotypes in health and disease. It was these aggregated features that allowed us to visualize the spectrum of male and female phenotypes in transcriptomic space.

**Fig. 5:**
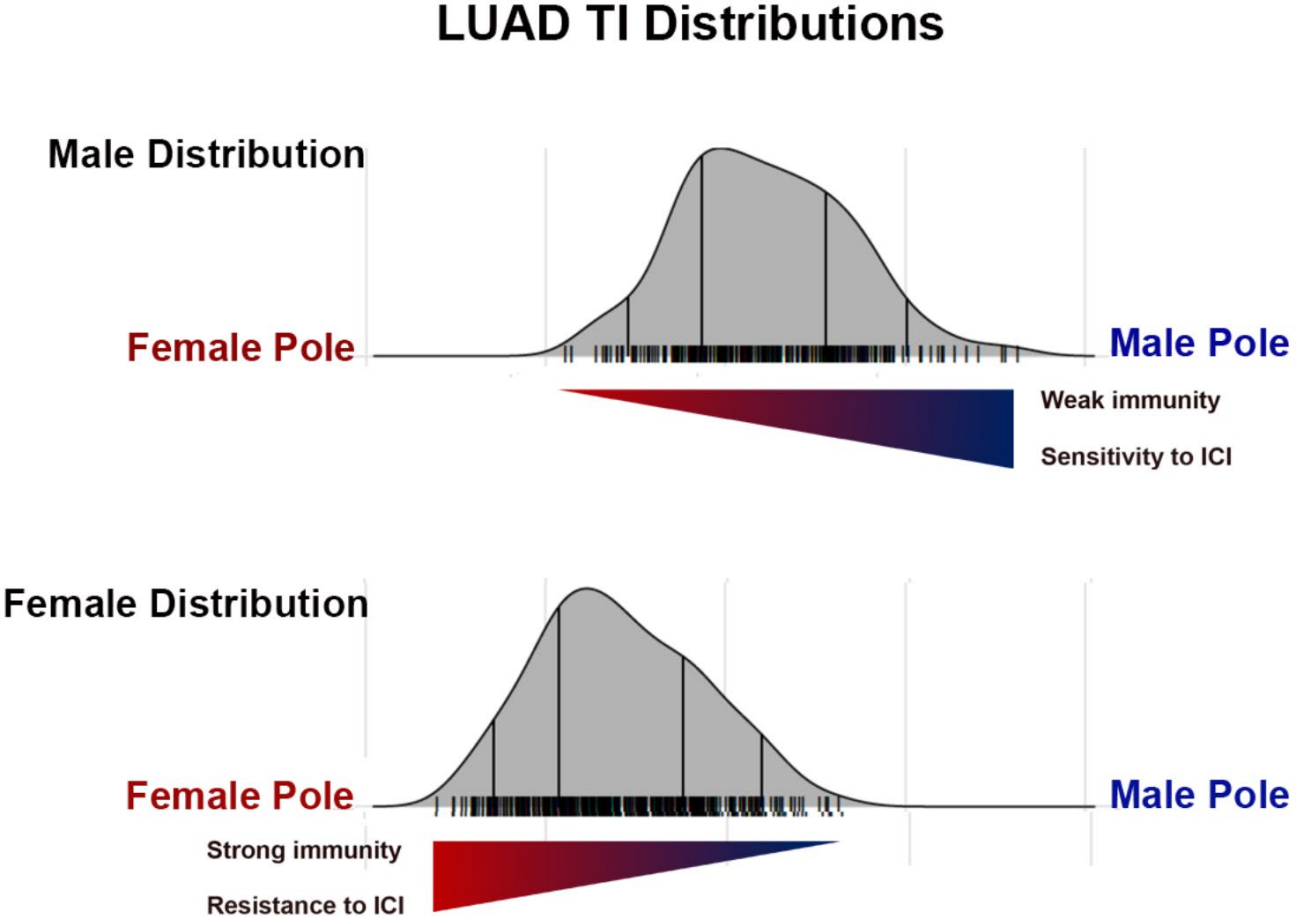
Example application of TI in patient treatment stratification. Pictured are the TI distributions for female (top panel) and male (bottom panel) lung adenocarcinoma (LUAD) patients. Females exhibited a strong immunity/inflammation signature and in clinical trials, are resistant to immune checkpoint inhibition (ICI). In contrast, male LUAD patients do not exhibit an inflammation/immunity signature and are responsive to ICI. If male and female patients were stratified for immune checkpoint inhibition treatment, the most likely males to respond to treatment would be those with the highest TI, those nearest the male pole. Female patients most likely to respond to treatment would also be those with the highest TI values, those furthest from the female pole

We observed several different polarization patterns, highlighting that sex and gender interact in variable ways with differing cancer mechanisms, i.e., cells of origin, specific oncogenic events, as well as their tissue and systems biology. The most common polarizations occur around cell cycle regulation and inflammation/immunity in both adult and pediatric cancers. This serves as validation for this approach as both mechanisms are already known to exhibit substantial sex differences [11–14]. As both pathways are targetable with available therapeutics [31, 32], it is interesting to consider how the TI approach might inform stratification for treatment. As an illustration, clinical experience indicates that females exhibit a smaller survival benefit from immune checkpoint inhibition (ICI) than males [33–35]. Thus, it would be reasonable to hypothesize that the immune signature associated with the lowest TI values is one of resistance to ICI. If so, then females with low TI values would be predicted to be less responsive to ICI than those with higher TI values. Concordantly, males have been shown to be more responsive to ICI and therefore, high TI values may be a biomarker for ICI sensitivity and males with lower TI values may be more resistant to ICI than those with higher TI values (Fig. 5). In this way, sex, cancer type, and TI values might more precisely stratify patients for ICI, or by analogy, other targetable pathways.

We can no longer focus on questioning whether sex and gender differences matter in cancer. Sex and gender differences in incidence, response to standard treatments, and survival, all strongly argue they do. The focus should be on how we use the continuously varying nature of sex and gender differences for treatment planning, patient stratification, and analysis of laboratory and clinical research results. As the TI analysis enriches patient-specific phenotyping, we expect that its use can enhance personalized approaches to treatment by realistically accounting for sex and gender effects in cancer. Importantly, substantial sex and gender differences exist in aging [36] and multiple conditions such as cardiovascular, rheumatological, psychiatric, and other diseases with substantial impact on the human condition [37]. The TI approach is readily adaptable to these conditions as well.

It is also important to note that the nature and magnitude of sex differences vary across the lifespan from pre- to post-, X inactivation, gonad formation and secretion of sex hormones, pubescence, and menopause. In addition, post-puberty, cancer rates rise as an exponential function of age. Thus, cancer TI value distributions and their underlying programs of gene expression will likely change as a function of age and future, deeper phenotyping efforts will need to incorporate age and a more informed understanding of how genetic ancestry, culture, and other social determinants of health impact on cancer phenotypes.

There are limitations to this study that can be addressed in future work. Validation of results for each cancer-type could be obtained through additional analyses of datasets such as the Chinese Cancer Genome Atlas. Importantly, retrospective analyses of relations between TI values and targeted treatment responses could provide important justification to test the TI approach in prospective clinical trials.

Finally, we need to address the ambiguities and inequities that arise with terms like male, female, men, women, alone or when combined with terms like -specific, -differences, -biased, -effect, etc. While we have used these terms, it is in part because we have imposed the common sex dichotomy in defining our poles. Available clinical data makes this practical, but it is not required. Any important variable such as carbohydrate- versus fatty acid- centered metabolism exhibits variation across the population. While this variation is visualizable along the sex and gender axis, this is a correlation and does not identify causation. We showed sex differences in cancer cell metabolism is correlated with sex differences in response to agents that target metabolism [9]. This is clinically important information but does not mean there is a male versus a female metabolism. As we gain a greater understanding of why elements of metabolism and other traits aggregate around male and female poles, we will be able to move away from analyses that rely on categorical sex as a hierarchical organizing/causative force for complex phenotypes. Instead, we can focus on where between poles of carbohydrate and fatty acid weighted metabolisms, an indivudal lies.

### Perspectives and significance

We must work to understand the basis for the significant sex and gender differences that exist in cancer mechanisms, incidence, and survival. This understanding will be necessary to incorporate sex and gender differences into personalized cancer treatments. As sex and gender is not a dichotomous variable, sex and gender-specific treatments are unrealistic. The TI approach described here represents a substantial advance in analyzing sex and gender effects in disease, one that more realistically treats sex and gender as a continuous, rather than, categorical variable. Deployment of this stratification tool could have a substantial impact on the personalization of cancer treatments.

## Conclusions

More realistic approaches to using sex and gender differences in personalized cancer treatments are possible when treating sex and gender as a continuous variable.

**Table 1:** Case Data

PANCAN Types	genes	Male	Female	Total	% Male
Thyroid Carcinoma (THCA)	20,531	157	415	572	27.4
Adrenal Cortical Carcinoma (ACC)	20,531	31	48	79	39.2
Pheochromocytoma and Paraganglioma (PCPG)	20,531	84	103	187	44.9
Sarcoma (SARC)	20,531	120	145	265	45.3
Diffuse Large B-Cell Lymphoma (DLBC)	20,531	22	26	48	45.8
Lung Adenocarcinoma (LUAD)	20,531	265	311	576	46.0
Cholangiocarcinoma (CHOL)	20,531	22	23	45	48.9
Thymoma (THYM)	20,531	63	59	122	51.6
Colon Adenocarcinoma (COAD)	17,507	257	235	492	52.2
Rectal Adenocarcinoma (READ)	17,507	90	80	170	52.9
Acute Myeloid Leukemia (LAML)	16,765	93	80	173	53.8
Low Grade Glioma (LGG)	20,531	291	238	529	55.0
Pancreatic Adenocarcinoma (PAAD)	20,531	101	82	183	55.2
Uveal Melanoma (UVM)	20,531	45	35	80	56.3
Kidney Chromophobe (KICH)	20,531	52	39	91	57.1
Glioblastoma 525 (GBM525)	8720	320	205	525	61.0
Skin Cutaneous Melanoma (SKCM)	20,531	293	180	473	61.9
Glioblastoma (GBM)	20,531	107	59	166	64.5
Stomach adenocarcinoma (STAD)	16,765	291	159	450	64.7
Kidney Renal Clear Cell Carcinoma (KIRC)	20,531	398	208	606	65.7
Liver Hepatocellular Carcinoma (LIHC)	20,531	280	143	423	66.2
Bladder Carcinoma (BLCA)	20,531	311	116	427	72.8
Kidney Renal Papillary Cell Carcinoma (KIRP)	20,531	236	87	323	73.1
Head and Neck Squamous Cell Carcinoma (HNSC)	20,531	415	151	566	73.3
Lung squamous cell carcinoma (LUSC)	20,531	408	144	552	73.9
Mesothelioma (MESO)	20,531	71	16	87	81.6
Esophageal Carcinoma (ESCA)	19,076	165	31	196	84.2
Total (PANCAN)		4668	3213	7881	59.2
**Pediatric Types**	**genes**	**Male**	**Female**	**Total**	**% Male**
Neuroblastoma	22,586	104	105	209	49.8
ATRT	25,076	12	12	24	50.0
Craniopharyngioma	25,076	19	15	34	55.9
DNET	25,076	13	9	22	59.1
Ependymoma	25,076	44	32	76	57.9
Ganglioglioma	25,076	24	17	41	58.5
High grade glioma	25,076	43	56	99	43.4
Low grade glioma	25,076	116	98	214	54.2
Medulloblastoma	25,076	68	37	105	64.8
PNET	25,076	6	11	17	35.3
Others	25,076	128	100	228	56.1
Total Pediatric		577	493	1069	54.0

### Electronic supplementary material

Below is the link to the electronic supplementary material.

**Supplementary Figure 1:** Pan Cancer Ridge Plots.

**Supplementary Figure 2:** Pediatric Cancer Ridge Plots.

**Supplementary Table 1:** Median TI and IRR.

**Supplementary Table 2:** Pan Cancer Gene Lists.

**Supplementary Table 3:** KF Database Genes.

**Supplementary Table 4:** CBTN Database Genes.

**Supplementary Table 5:** Shared KF and CBTN Genes.

## Data Availability

The results analyzed and published here are based upon datasets generated by the TCGA Research Network https://www.cancer.gov/tcga, accessed from https://pancanatlas.xenahubs.net, by Gabriella Miller Kids First Pediatric Research Program projects (phs001436.v1.p1), accessed from dbGaP (www.ncbi.nlm.nih.gov/gap), and by the Childhood Brain Tumor Tissue Consortium, accessed from https://cbtn.org.

## Code availability

The R package for the Bayesian Nearest Neighbors method could be accessed from https://github.com/wuwill/BKNN.
