# Supplementary material for: Treating sex and gender differences as a continuous variable can improve precision cancer treatments
Source: Biol Sex Differ. 2024 Apr 15;15:35. doi: 10.1186/s13293-024-00607-1 (PMC11017567; doi:10.1186/s13293-024-00607-1)
Supplement: Supplementary file 2 — Supplementary Figure 2: Pediatric Cancer Ridge Plots. [file 13293_2024_607_MOESM2_ESM.pdf]

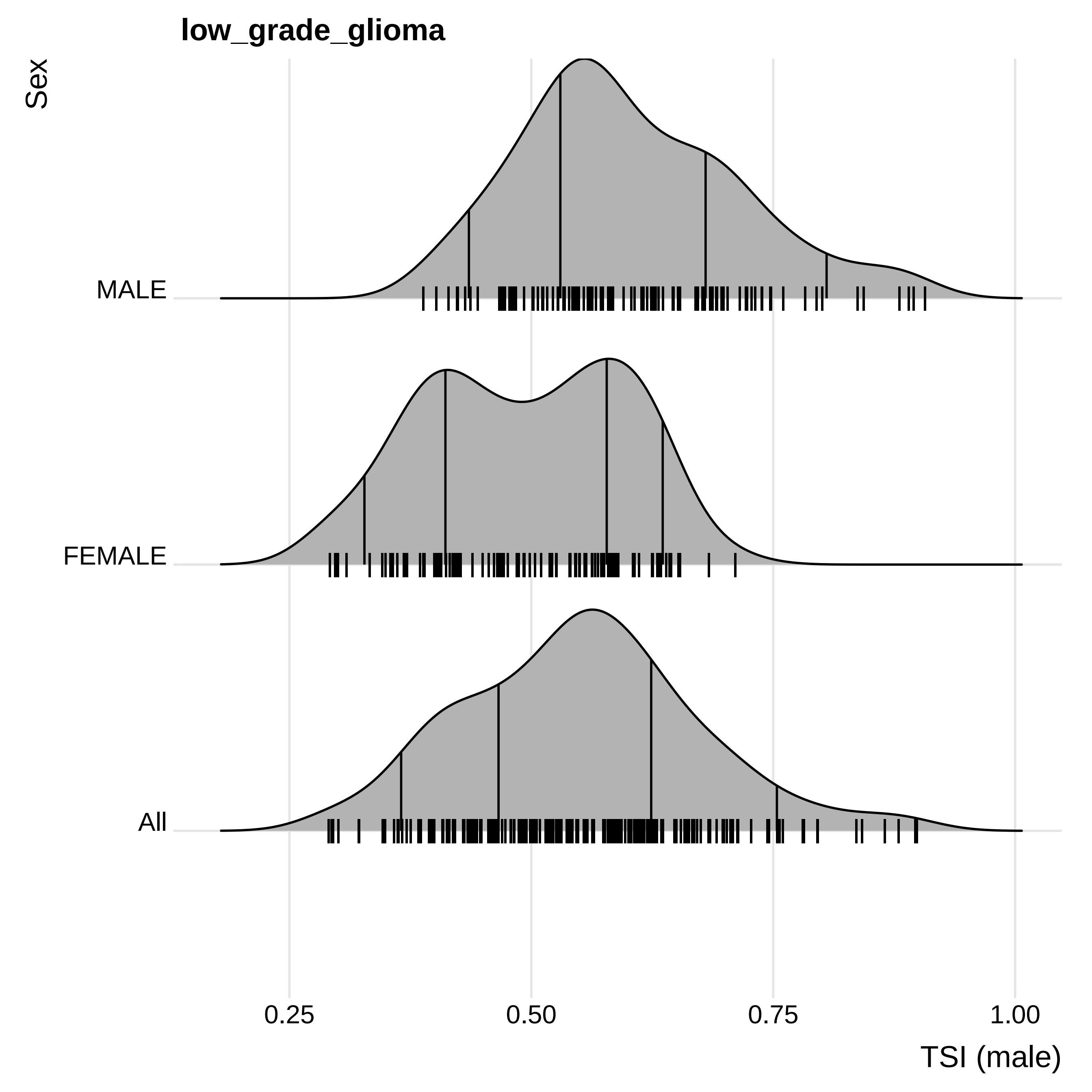
low_grade_glioma
Sex
MALE
FEMALE
All
0.25
0.50
0.75
1.00
TSI (male)

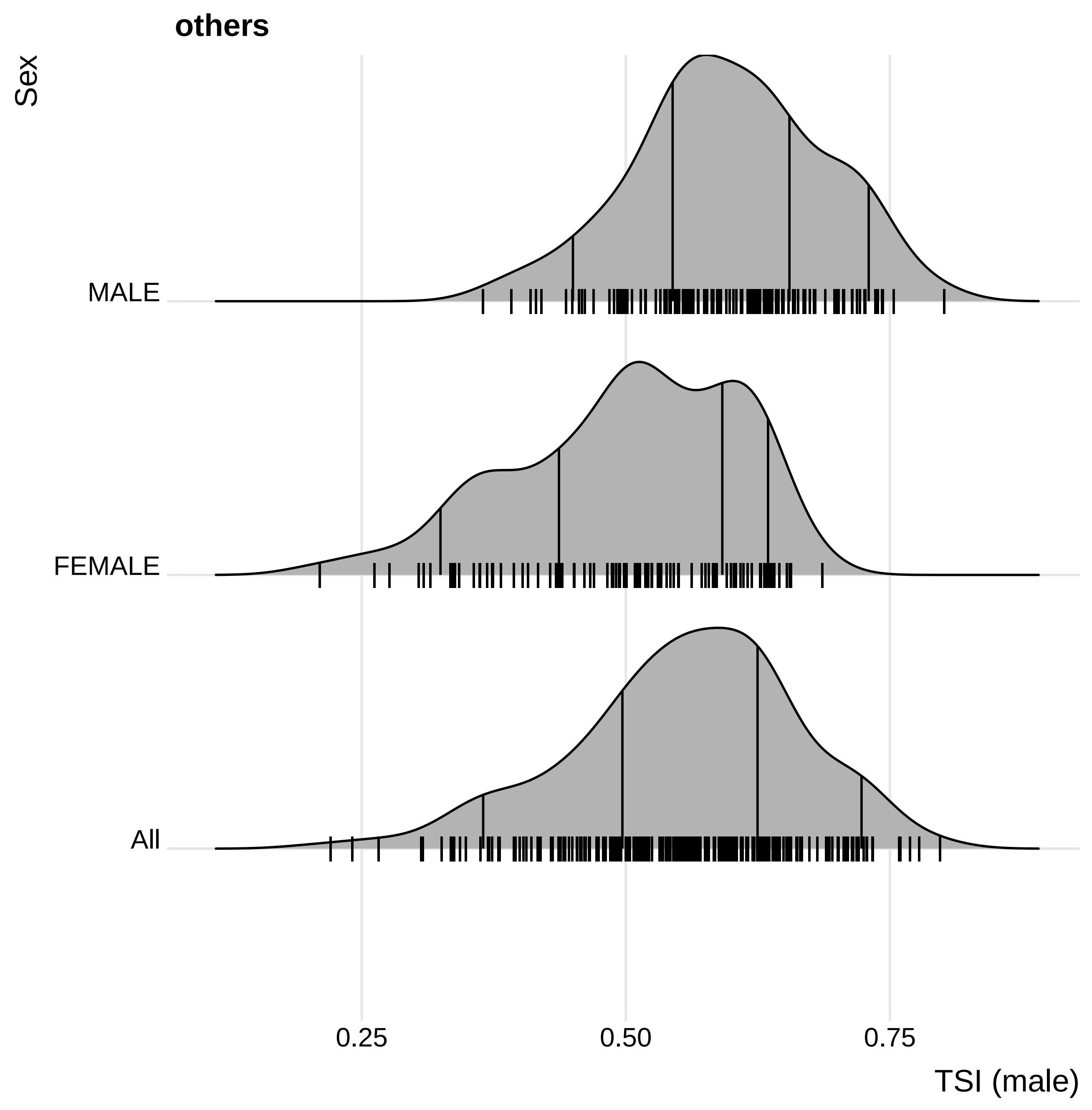

others
Sex
MALE
FEMALE
All
0.25
0.50
0.75
TSI (male)

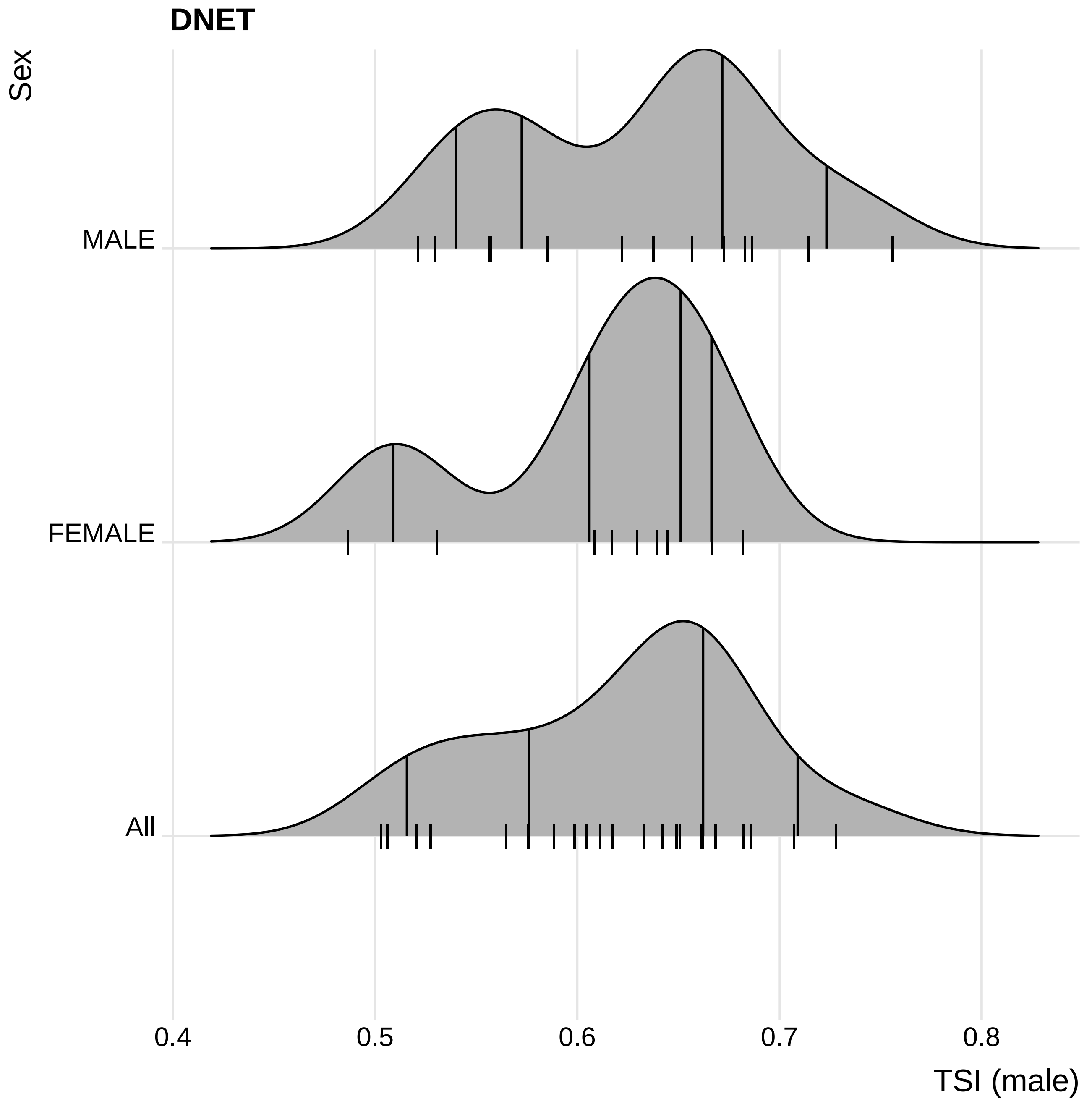
DNET
Sex
MALE
FEMALE
All
0.4
0.5
0.6
0.7
0.8
TSI (male)

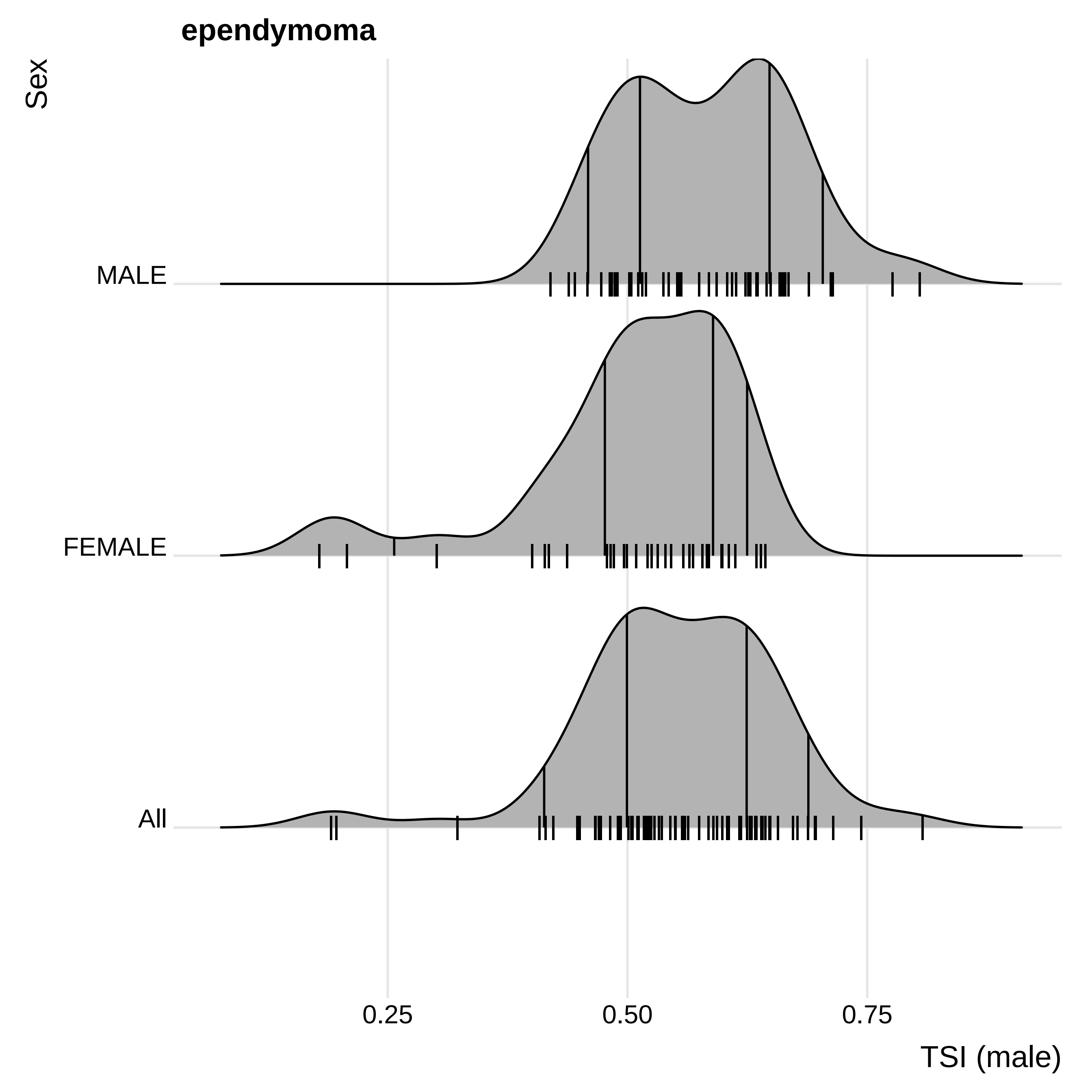
ependymoma
Sex
MALE
FEMALE
All
0.25
0.50
0.75
TSI (male)

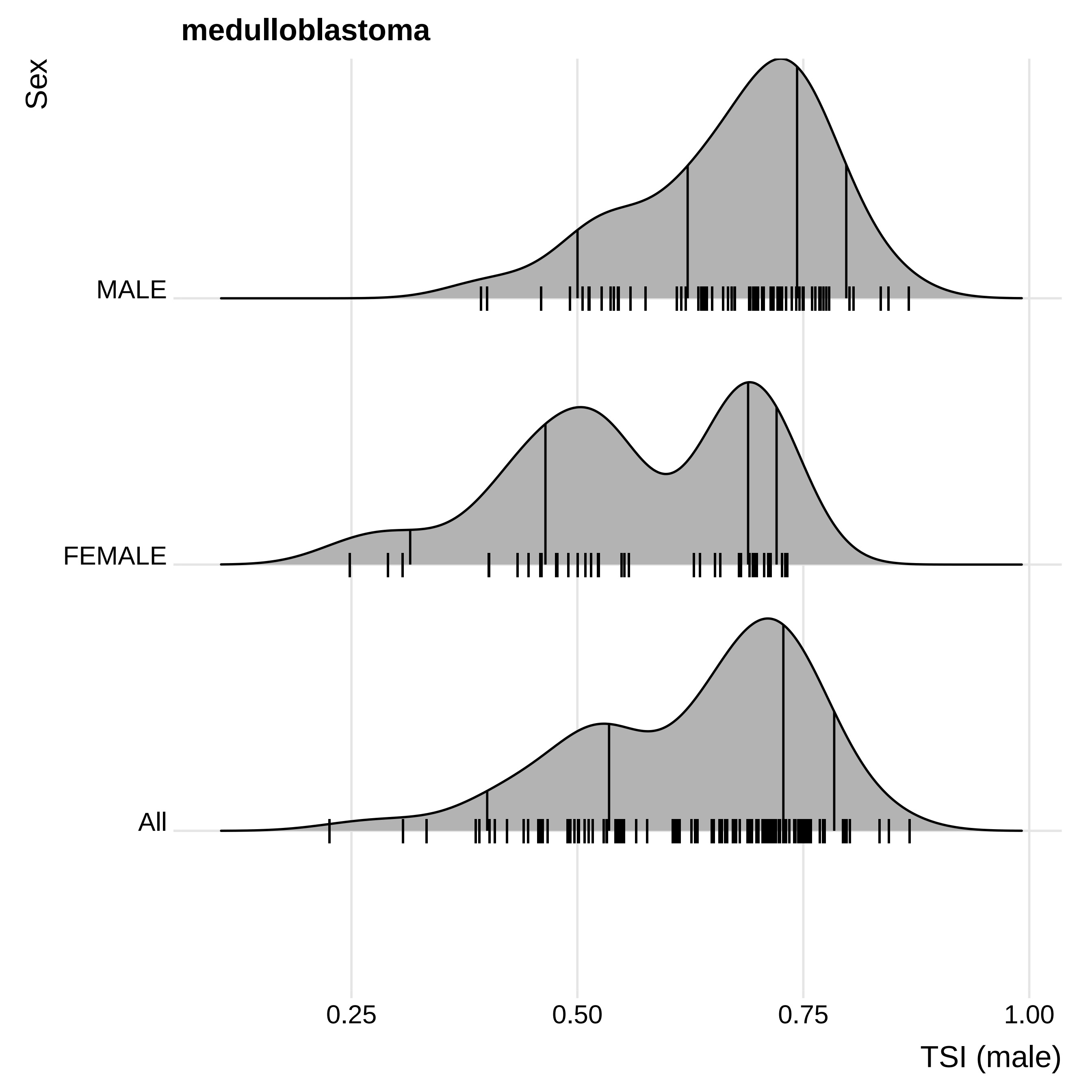
medulloblastoma
Sex
MALE
FEMALE
All
0.25
0.50
0.75
1.00
TSI (male)

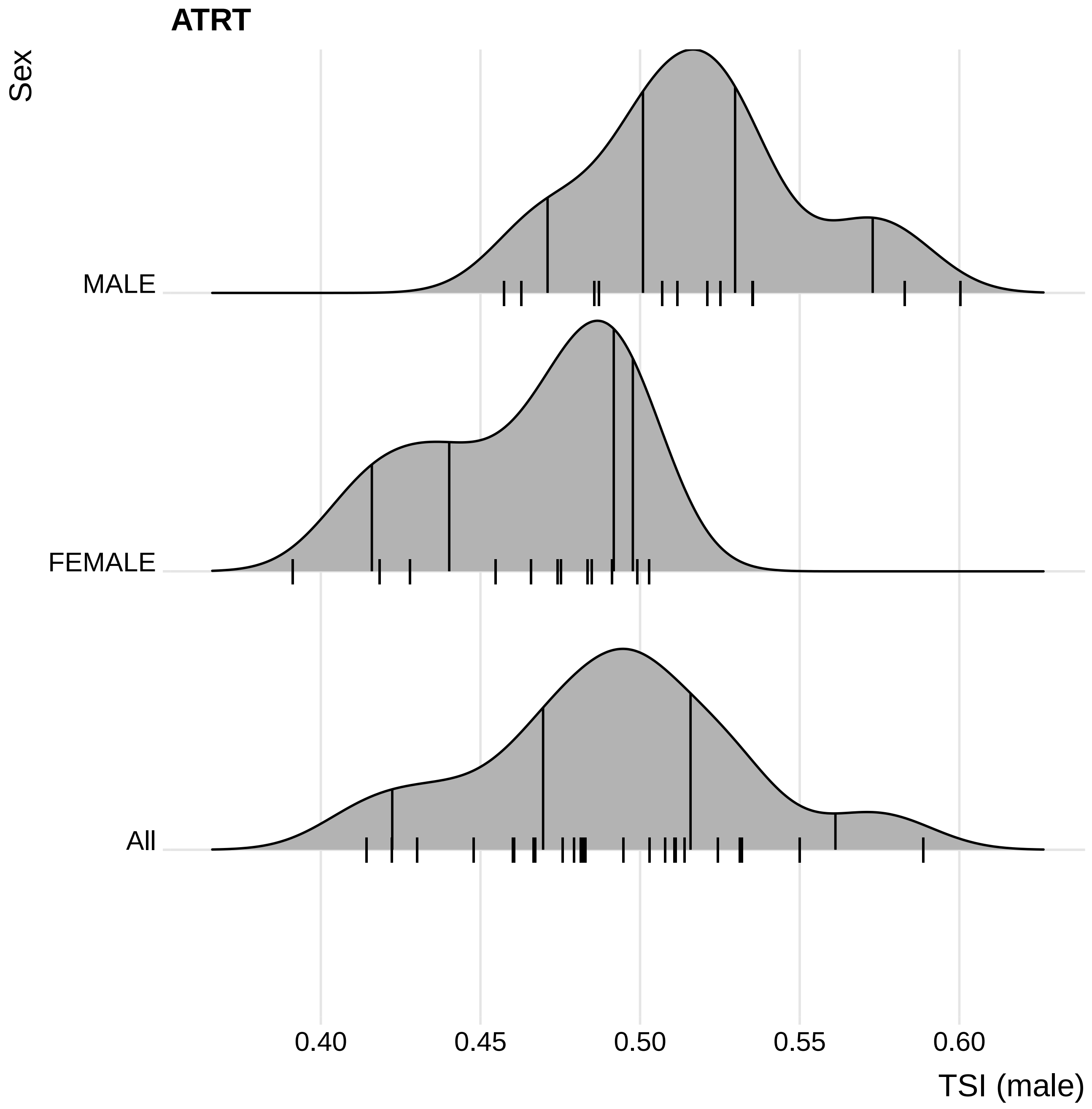
ATRT
Sex
MALE
FEMALE
All
0.40
0.45
0.50
0.55
0.60
TSI (male)

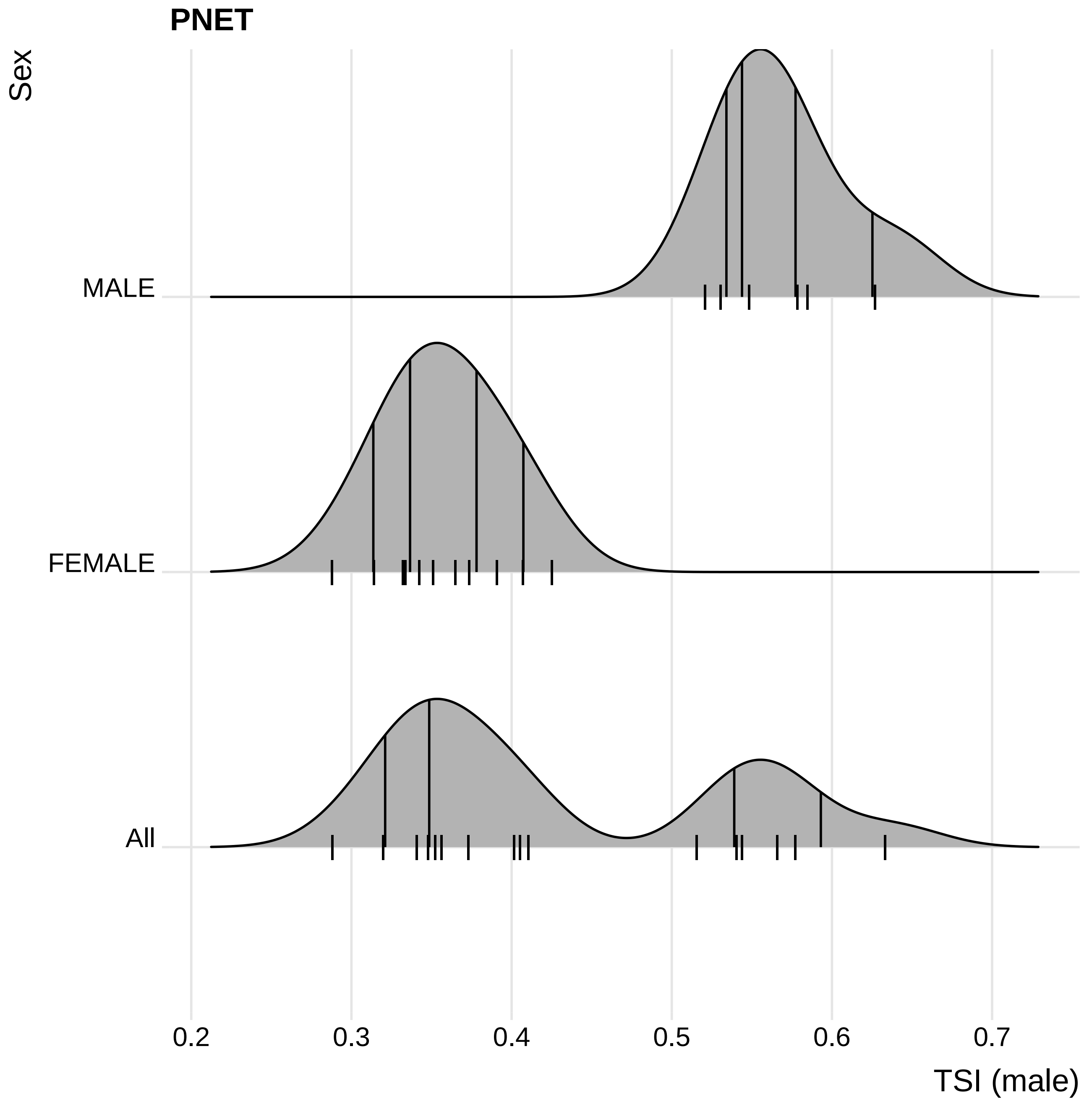

PNET
Sex
MALE
FEMALE
All
0.2
0.3
0.4
0.5
0.6
0.7
TSI (male)

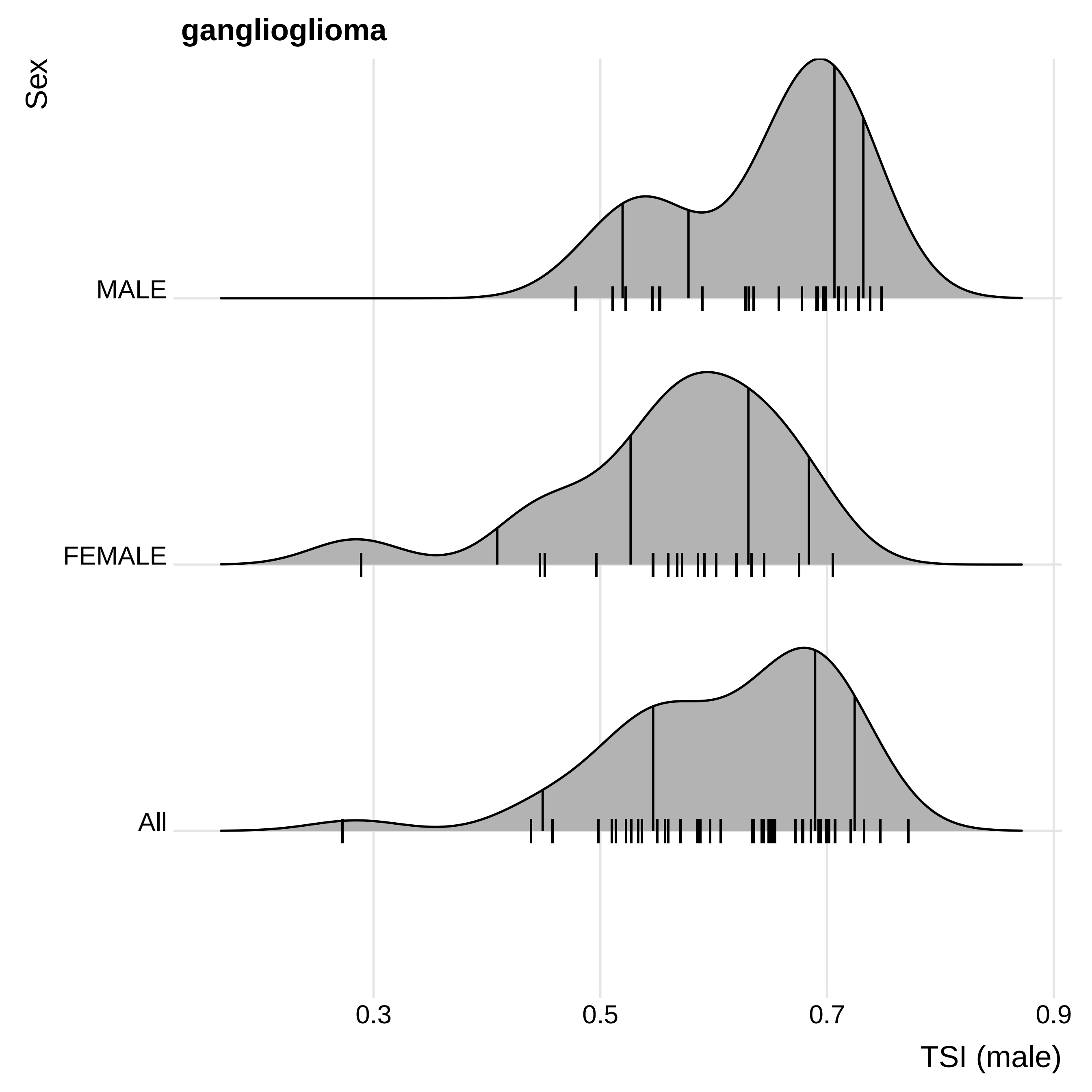
ganglioglioma
Sex
MALE
FEMALE
All
0.3
0.5
0.7
0.9
TSI (male)

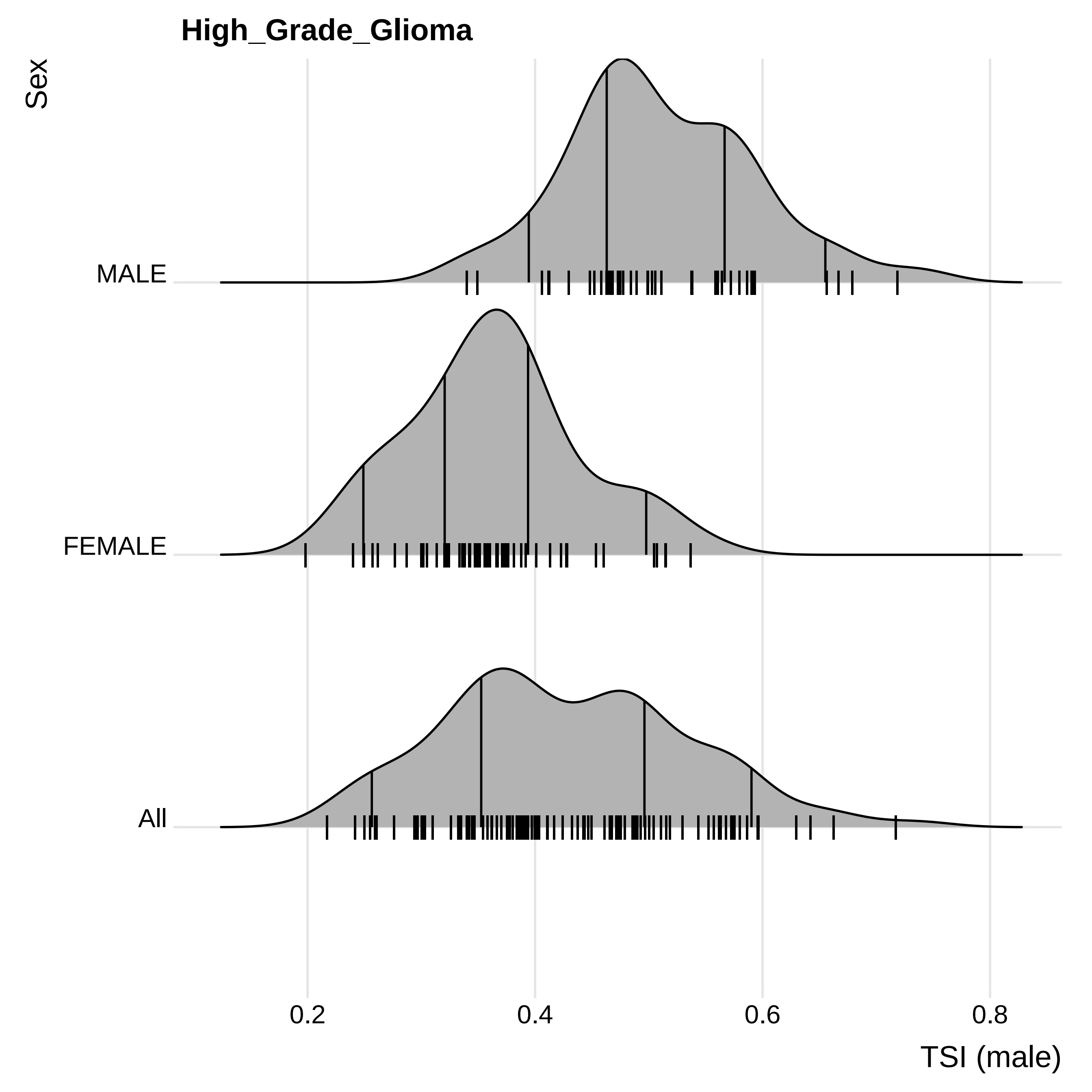
High_Grade_Glioma
Sex
MALE
FEMALE
All
0.2
0.4
0.6
0.8
TSI (male)

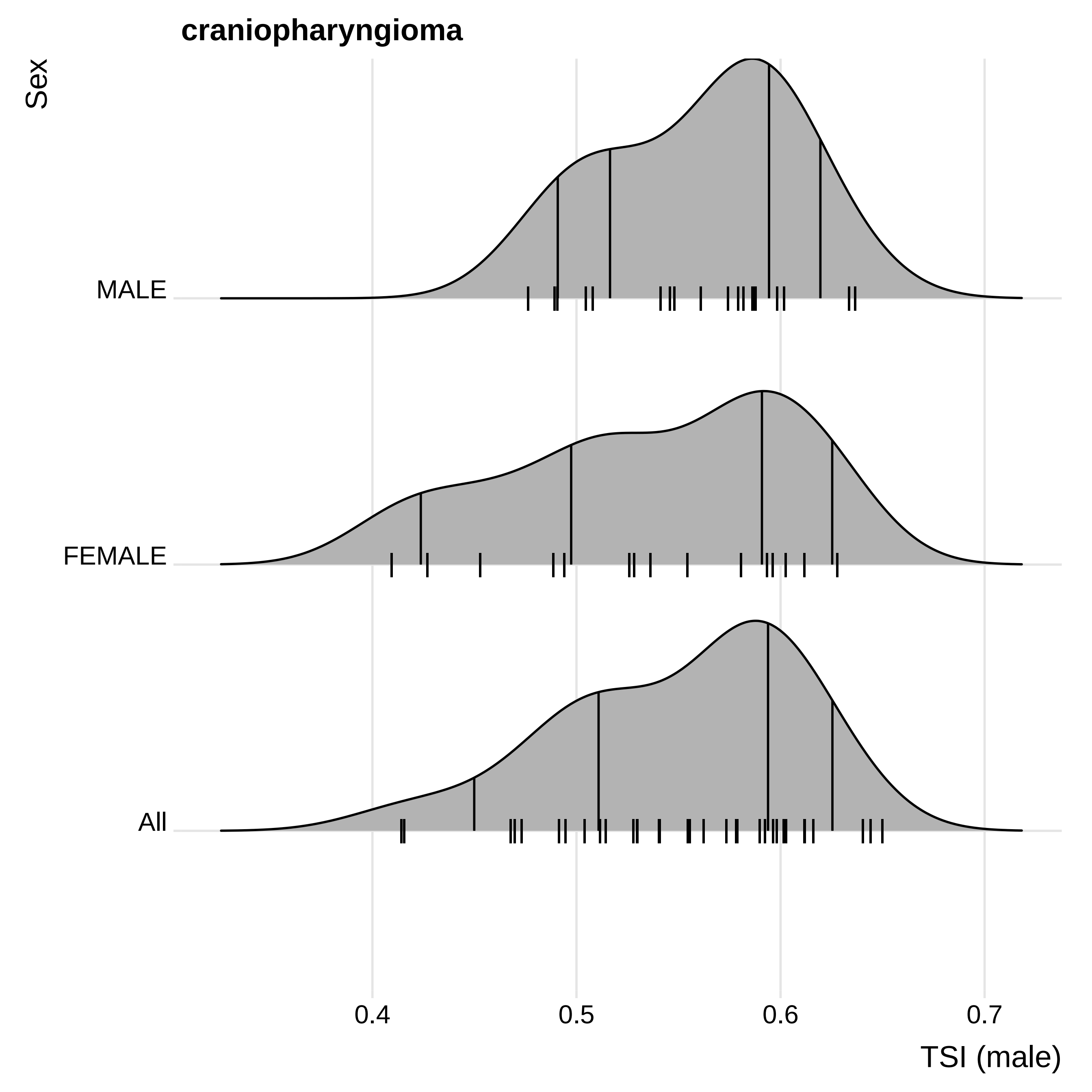

craniopharyngioma
Sex
MALE
FEMALE
All
0.4
0.5
0.6
0.7
TSI (male)

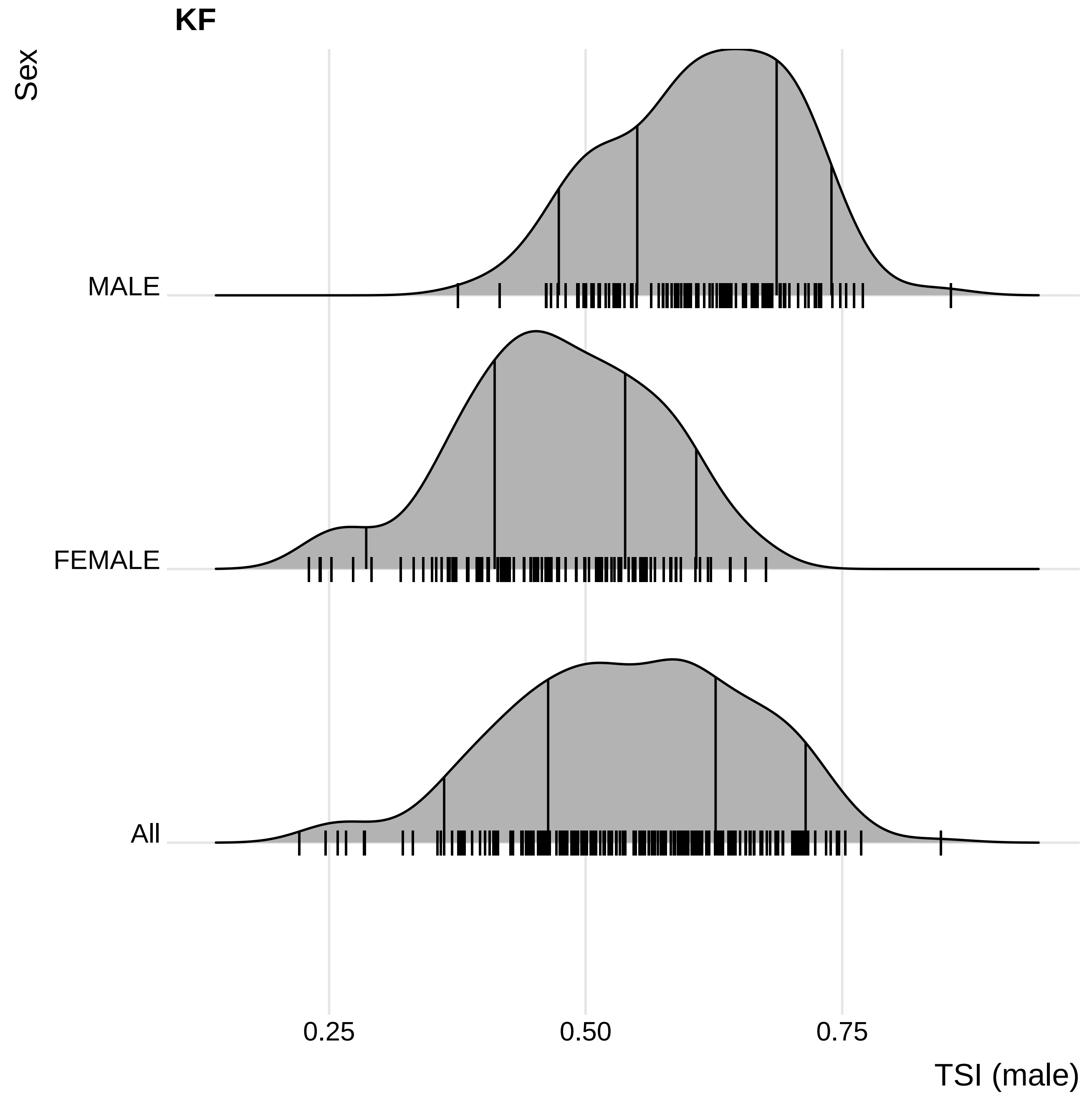
KF
Sex
MALE
FEMALE
All
0.25
0.50
0.75
TSI (male)
